# Balancing sterilization and functional properties in Poloxamer 407 hydrogels: comparing heat and radiation techniques

**DOI:** 10.1093/rb/rbaf005

**Published:** 2025-01-22

**Authors:** Angela De Lauretis, Anne Eriksson Agger, Antara Pal, Jan Skov Pedersen, Szymon Mikolaj Szostak, Reidar Lund, Ståle Petter Lyngstadaas, Jan Eirik Ellingsen, Dirk Linke, Håvard Jostein Haugen

**Affiliations:** Department of Biomaterials, Institute of Clinical Dentistry, Faculty of Dentistry, University of Oslo, 0455 Oslo, Norway; Corticalis AS, Oslo Science Park, 0349 Oslo, Norway; Department of Biomaterials, Institute of Clinical Dentistry, Faculty of Dentistry, University of Oslo, 0455 Oslo, Norway; Interdisciplinary Nanoscience Center (iNANO), Aarhus University, 8000 Aarhus C, Denmark; Department of Chemistry, Aarhus University, 8000 Aarhus C, Denmark; Interdisciplinary Nanoscience Center (iNANO), Aarhus University, 8000 Aarhus C, Denmark; Department of Chemistry, Aarhus University, 8000 Aarhus C, Denmark; Department of Chemistry, University of Oslo, 0315 Oslo, Norway; Department of Chemistry, University of Oslo, 0315 Oslo, Norway; Hylleraas Centre for Quantum Molecular Sciences, University of Oslo, 0315 Oslo, Norway; Department of Biomaterials, Institute of Clinical Dentistry, Faculty of Dentistry, University of Oslo, 0455 Oslo, Norway; Department of Prosthetics and Oral Function, Institute of Clinical Dentistry, University of Oslo, 0455 Oslo, Norway; Department of Biosciences, University of Oslo, Oslo 0316, Norway; Department of Biomaterials, Institute of Clinical Dentistry, Faculty of Dentistry, University of Oslo, 0455 Oslo, Norway

**Keywords:** Poloxamer 407, hydrogels, sterilization, functional properties

## Abstract

Poloxamer 407, also known as Pluronic^®^ F127, is gaining interest in the cosmetic, biomedical and pharmaceutical fields for its biocompatibility, safety and thermo-sensitive properties. Ensuring sterility is critical in clinical applications, and sterilization is often preferred over aseptic processing. However, sterilization can impact the functional properties of the hydrogel. In this study, we investigate the effects of steam heat (121°C, 20 min), dry heat (160°C, 1 h), gamma irradiation (25 kGy) and electron beam (e-beam) irradiation (15 and 25 kGy) on a 30% w/v Poloxamer 407 hydrogel formulation. Our analysis encompasses gelling properties, pH, Fourier-transform infrared spectroscopy, gel permeation chromatography, small-angle X-ray scattering, rheology, swelling, degradation by-products and lactate dehydrogenase release of the sterilized hydrogels, comparing them to a non-sterile counterpart. We demonstrated that heat sterilization alters the hydrogel’s gelling and structural properties due to water evaporation and oxidation under harsh temperature conditions, especially when applying the dry heat method. Gamma irradiation proved unsuitable, resulting in an acidic and cytotoxic hydrogel due to oxidative degradation. In contrast, e-beam irradiation preserves the hydrogel’s elasticity, gelling and structural properties while enhancing mechanical resilience and moderating swelling. Therefore, e-beam irradiation within the 15–25 kGy range appears to be the most suitable method for sterilizing a 30% w/v Poloxamer 407 hydrogel.

## Graphical Abstract

**Figure:**
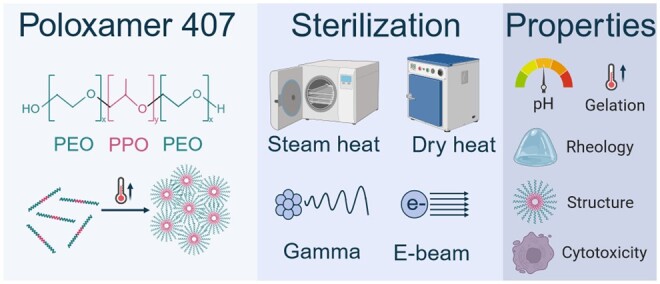

## Introduction

Poloxamer 407, also known as Pluronic^®^ F-127, is a water-soluble triblock copolymer consisting of a central hydrophobic polypropylene oxide (PPO) block flanked on both sides by hydrophilic polyethylene oxide (PEO) blocks [1–3]. With an average molecular weight of 12.6 kDa, PEO segments comprise 70% of its composition [1–3]. In aqueous solutions at low temperatures, Poloxamer 407 is found as monomers [3, 4]. However, as the temperature increases, the system undergoes a sol–gel transition, wherein the amphiphilic properties prompt self-aggregation into spherical micelles, featuring an outer shell of PEO units and an inner core of PPO units [1–6]. These nano-sized micelles, ranging from 10 to 200 nm, emerge at the critical micelle concentration (CMC) and critical micelle temperature (CMT) [1–5, 7]. The CMC decreases as the temperature rises, while an increase in Poloxamer 407 concentration lowers the CMT [1–5, 7]. Increasing the temperature, the micelles first rearrange into a cubic structure and then form a hexagonal structure [2, 6]. This ordered packing is macroscopically observed as a thermo-sensitive hydrogel in sufficiently concentrated samples [3–5]. Typically, a concentration of 15–20% w/v is necessary to achieve gelation at body temperature [8, 9].

Owing to their non-toxic, non-irritating and non-sensitizing characteristics together with their biocompatibility, low cytotoxicity, and thermo-sensitive behaviour, Poloxamer 407 hydrogels have attracted considerable interest in the cosmetic, biomedical and pharmaceutical sectors [8, 10–13]. Particularly, the thermo-sensitive properties ensure that the hydrogel transitions from a liquid to a gel at body temperature, making it ideal for applications where prolonged retention, controlled drug release, and structural stability are essential, such as in drug delivery systems and tissue regeneration. Indeed, these hydrogels find common applications in personal hygiene products, in solubilizing and delivering hydrophobic drugs, in gene delivery, and as scaffolds for tissue engineering [3, 4, 9, 11, 14–27]. Some formulations, including Poloxamer 407, have already received approval from the US Food and Drug Administration [28].

To allow for commercialization within the medical and pharmaceutical industries, sterility is crucial for adherence to safety guidelines outlined in the European Medical Device Regulation, by the European Medicines Agency, and the Food and Drug Administration, among others [29–31]. Within the regulatory framework, numerous ISO standards are adopted in the industry to ensure sterility, each specific to different methods such as aseptic processing, physical methods (heat, radiation, filtration) and chemical methods (e.g. using ethylene oxide, ozone, alcohols) [32]. Sterilization is preferred over aseptic processing due to the latter's need for an extremely controlled environment and higher costs [33]. However, careful consideration must be given during the sterilization processes, as materials may be sensitive to such procedures, risking degradation, discolouration and/or toxicity [34]. Sterilizing hydrogels is particularly challenging due to the presence of water, which can amplify unwanted effects, altering their properties [35–38]. Common sterilization methods for hydrogels include steam heat, dry heat, gamma irradiation and electron beam (e-beam) [34, 39]. Steam heat sterilization occurs in an autoclave at temperatures between 121°C and 130°C for 15–20 min, denaturing vital cell components to eliminate microorganisms [34, 39, 40]. Dry heat requires higher temperatures (160–190°C) and longer exposure times, in the range of hours, with microorganisms being inactivated through oxidation [34, 39, 40]. While fast and effective, heat sterilization might affect the structural properties of hydrogels by reducing their water content through evaporation [40].

Conversely, gamma rays and electron beams use ionizing radiation, which breaks microbial DNA and RNA chains and generates free radicals damaging cellular components [34, 39–41]. Gamma irradiation, sourced from ^60^Co or ^137^Cs with a dose of 10–30 kGy, and e-beam irradiation, produced by accelerating electrons with a dose dependent on the power source, are both easy to control but can be costly [34, 39–41]. While the standard irradiation dose is 25 kGy, other doses can be employed as long as the sterilization process is validated [34, 39]. Gamma irradiation offers higher penetration ability than e-beam but tends to induce greater changes in structural properties [34, 39–41].

Currently, limited information regarding the sterilization processes of Poloxamer 407 hydrogels is available [42, 43]. Before selecting the optimal sterilization method, assessing how this impacts the hydrogel properties is essential. Indeed, the hydrogel must preserve suitable characteristics to fulfil its intended purpose after sterilization, which is one of the major challenges for clinical translation. In the present study, we investigate the effects of sterilization through steam heat, dry heat, gamma irradiation and e-beam irradiation on the functional properties of a 30% w/v Poloxamer 407 hydrogel formulation.

## Materials and methods

### Materials

Poloxamer 407 (average *M_W_* = 12.6 kDa), Dulbecco’s phosphate-buffered saline (DPBS), tetrahydrofuran (THF, HPLC grade) and dimethylacetamide (DMAc, HPLC grade) were purchased from Merck Life Science AS, Norway. Polyethylene glycol (PEG) was purchased from Polymer Source Inc., Canada.

### Hydrogel production and sterilization

Poloxamer 407 hydrogels were produced via the cold method [44–46]. In short, with manual stirring, Poloxamer 407 was dissolved in Milli-Q water at a concentration of 30% w/v at 4°C. To ensure complete dissolution despite the high concentration of Poloxamer 407, the formulation was maintained at 4°C in a refrigerator for 24 h, rather than the typical overnight period. The dissolution was confirmed through visual inspection. Afterwards, the hydrogels underwent a single sterilization cycle through various methods: steam heat using a SX-700E Autoclave (Tomy, Japan) at 121°C for 20 min, dry heat in a L3/11 Nabertherm high-temperature oven equipped with a P320 controller (Nabertherm, Germany) at 160°C for 1 h, gamma irradiation at 25 kGy ± 10%, e-beam irradiation at 15 kGy ± 20% and e-beam irradiation at 25 kGy ± 20% (Synergy Health Radeberg GmbH, STERIS, Germany). The sterilization parameters were selected to align with industry-standard practices.

To maintain sterility, the samples were kept in a sealed container in a refrigerator, opened only before analysis. The maximum storage duration in a sterile state was 4 months.

### Gelling temperature and time

Sterilized and non-sterile hydrogels with a volume of 2 ml were introduced into vials and maintained at 15°C, an initial temperature chosen to keep the formulation in a liquid state for effective observation of gelling behaviour. The temperature was incrementally raised by 1°C, with vials examined every 2 min by vial inversion to ensure uniform temperature throughout the hydrogel. The gelation temperature was defined as the temperature at which the hydrogel ceased to flow. The experiment was conducted in triplicate.

### pH measures

The pH of the sterilized hydrogels at 4°C was determined using a PHM210 Standard pH metre (MeterLab^®^, Radiometer Analytical SAS, France) equipped with a pH sensor from VWR Chemicals, US. The pH values were compared to those of a non-sterile sample. The measurements were recorded in triplicate for each sample.

### Fourier-transform infrared spectroscopy

Fourier-transform infrared spectroscopy (FTIR, Spectrum 400, PerkinElmer, US) was used to analyse the infrared spectra of both non-sterile and sterile samples. The hydrogels were freeze-dried (LyoQuest, Telstar, Spain) before the spectra were recorded at room temperature within the 650 cm^−1^ − 4000 cm^−1^ range, employing a resolution of 4 cm^−1^ and recording 64 scans per spectrum.

### Swelling study

In the swelling study, 0.5 ml of hydrogel was introduced into a vial, weighed, and placed in a TS8056 incubator (Termarks, Sweden) at 37°C for 15 min. This temperature simulates body temperature for evaluating the hydrogel’s behaviour under clinically relevant conditions. At the same time, the 15-min duration was selected to ensure the formulation reached the gel state before the swelling test. Subsequently, 1.5 ml of DPBS at a concentration of 2.5% v/v was added to the vial and maintained at 37°C for 1, 3 and 6 h. After the specified incubation period, DPBS was removed from the vial, and weight changes were assessed to evaluate the swelling percentage. The study was conducted with five samples of each hydrogel. A one-tailed homoscedastic *t*-test was used to ascertain any statistically significant changes between the samples’ initial and final time points.

### Rheology

The rheological properties of the hydrogels were investigated through amplitude sweeps (shear strain: 0.01–100%; angular frequency: 10 rad/s) and frequency sweeps (shear strain: 0.05%; angular frequency: 0.1–100 rad/s) at a temperature of 37°C through a Peltier temperature controlled device (H-PTD 220), using an Anton Paar MCR 702e rheometer (Anton Paar, Austria) and a smooth 12-mm parallel plate (PP12) at a 1-mm gap.

### Gel permeation chromatography

The molecular weight of the hydrogels was determined using gel permeation chromatography (GPC) (HLC-8320GPC EcoSEC, Tosoh Co. Ltd, Japan) equipped with a refractive index detector (MiniDAWN TREOS, Wyatt Technology, US). The analysis was conducted at a standard temperature of 35°C with a 4.6 × 150 mm polystyrene-divinylbenzene TSKgel SuperHZ4000 column (Tosoh Co. Ltd, Japan). An 85:15 mixture of THF and DMAc served as the eluent at a flow rate of 0.35 ml/min, while PEG (2.1 kDa < *M_w_* < 23.8 kDa) dissolved in THF:DMAc (85:15) at a concentration of 5 mg/ml was used to prepare a standard calibration curve. The hydrogels were freeze-dried (LyoQuest, Telstar, Spain) and re-dissolved in THF:DMAc (85:15) at a 5 mg/ml concentration. Both the standards and samples were filtered through a 0.2-µm Titan3™ polyvinylidene fluoride syringe filter (Thermo Fisher Scientific, US) before analysis, and the injection volume was set at 20 µl. All measurements were performed in triplicate. The HLC-8320 EcoSEC System Control version 1.14 (Tosoh Co. Ltd, Japan) and ASTRA^®^ version 6.1.2.84 (Wyatt Technology, US) software were utilized for instrument operation and data acquisition.

### Small-angle X-ray scattering

Small-angle X-ray scattering (SAXS) was performed on a modified version of a NanoSTAR (Bruker AXS) pinhole camera installed on a rotating anode X-ray source (MacScience Cu with a 0.1 × 0.1 mm effective source size operated at 1 kW) with a 2D position-sensitive gas detector (Våntec 500). The instrumental details are described elsewhere [47, 48]. Scattering intensities are given on an absolute scale as a function of the modulus of the scattering vector *q* (*q *=* *4πsin(θ)/λ, where 2θ is the scattering angle and λ is the wavelength of the incoming X-rays). The scattering intensity (*I*_(*q*)_) was recorded at a low-*q* setup with a sample-to-detector distance of 106 cm giving a *q* vector range of 0.0045–0.23 Å^−1^ and can generally be described by: $I\left(q\right)=\Delta \rho^{2}\varphi V_{p}S\left(q\right)P\left(q\right)+I_{\mathrm{background}}$, where *Δρ* is the difference in scattering length density between the scattering particle and the solvent, *φ* is the volume fraction of the scattering particle, *V_p_* is the volume of a single scattering particle, *S*_(*q*)_ is the inter-particle structure factor, *P*_(*q*)_ is the form factor and *I*_background_ is the background. Since the samples were solid at room temperature, they were cooled to 4°C to liquify and to fill the reusable capillary, which was used both for samples and background measurements. All the samples were measured at room temperature (20°C), and the data were subtracted from the background using scattering from Milli-Q water.

### LDH assay

A murine osteoblast precursor cell line, MC3T3-E3 (ATCC, Manassas, VA, US), was used to investigate the cytotoxicity of the hydrogels. Cells were cultured in minimum essential medium alpha, MEM α (A10490-01; Gibco, Waltham, MA, US) supplemented with 10% fetal bovine serum (10270-106, Gibco), 100 U/ml penicillin and 100 µg/ml streptomycin (15140-122; Gibco) in a humidified atmosphere of 5% CO_2_ at 37°C. Cells were seeded at 30 000 cells/cm^2^ density in culture-treated 48-well plates.

To investigate the cytotoxic effects of the hydrogels, 1 ml of each hydrogel was allowed to solidify for 10 min before an equal amount of cell culture medium (1 ml) was added for 24 h. Two hundred microlitres of the conditioned medium was added to the cells in triplicate. After 24 h, 50-µl medium was collected and the medium was exchanged with a new conditioned medium for an additional 24 h, after which 50-µl media was collected. Cells exposed to fresh culture medium were used as controls.

Following the manufacturer’s description, cytotoxicity was evaluated using a lactate dehydrogenase (LDH) activity kit (11644793001; Roche, Basel, Switzerland). In short, 50 µl of the collected media was mixed with 50 µl of the reaction mixture and incubated at room temperature in the dark for 30 min. The absorbance was measured at 490 nm using a BioTek ELx800 Absorbance Microplate Reader (BioTek Instruments, Inc., Winooski, VT, US). LDH activity was measured after 24 and 48 h. Results were normalized to controls and analysed using a two-tailed Student’s *t*-test in SigmaPlot version 14 (Systat Software, San Jose, CA, US).

### Determination of peroxide groups

The Pierce™ Quantitative Peroxide Assay Kit—Aqueous Compatible Formulation (Thermo Fisher Scientific, US) was used to examine and quantify the formation of peroxide groups resulting from possible degradation reactions during the sterilization processes. Hydrogen peroxide standard dilutions were prepared according to the manufacturer’s instructions. The absorbance was measured at 562 nm using a BioTek ELx800 Absorbance Microplate Reader (BioTek Instruments, Inc., Winooski, VT, US).

### Determination of carbonyl groups

A MAK486 Carbonyl Assay Kit (Merck Life Science AS, Norway) was used to examine and quantify the formation of carbonyl groups resulting from possible degradation reactions during the sterilization processes. Carbonyl standard dilutions were prepared according to the manufacturer’s instructions, while the hydrogel samples were diluted 1:20 with Milli-Q water. The absorbance was measured at 405 nm using a BioTek ELx800 Absorbance Microplate Reader (BioTek Instruments, Inc., Winooski, VT, US).

### Statistics

For pH measurements, swelling studies, molecular weight as determined by GPC, LDH assay and the quantification of peroxide and carbonyl groups, one-way analysis of variance (ANOVA) was performed with GraphPad Prism version 10.2 (GraphPad Software Inc., San Diego, CA, US) to identify any statistically significant differences between the groups.

## Results

**Figure 1.**
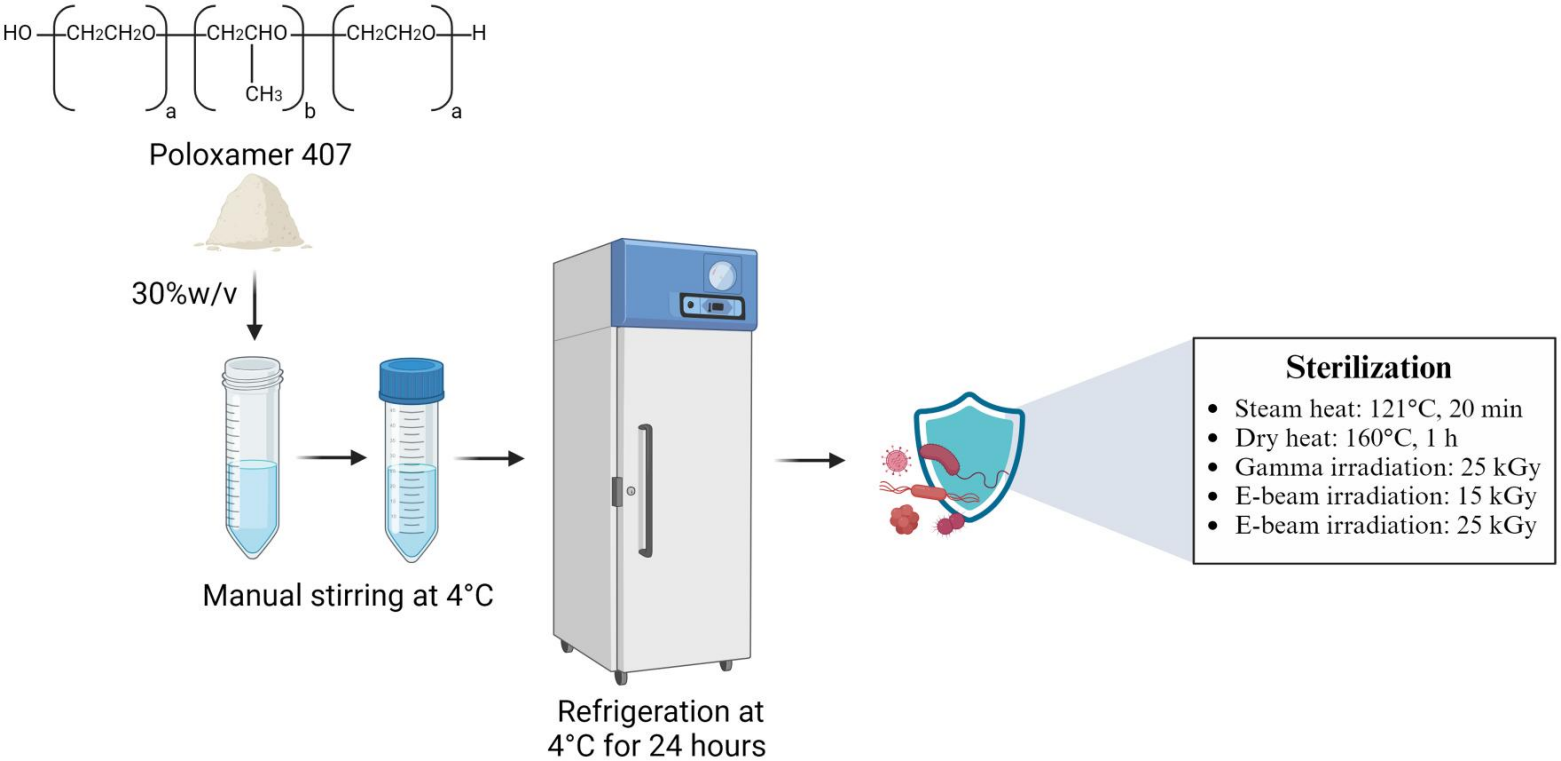
30%w/v Poloxamer 407 hydrogel formulation and sterilization processes.

We prepared a hydrogel formulation with a composition of 30% w/v Poloxamer 407, which underwent sterilization through various methods: steam heat (121°C, 20 min), dry heat (160°C, 1 h), gamma irradiation (25 kGy) and e-beam irradiation (15 and 25 kGy). The process is visually summarized in Figure 1. Subsequently, we assessed the hydrogels and their functional properties and compared them to a non-sterile sample.

### Gelling time, temperature and pH

Gelling time and temperature were used to assess changes in the gelation process before and after sterilization (Table 1). All the samples gelled within 2 min at the corresponding gelling temperature. Remarkably, steam-heated and e-beam sterilized samples maintained an unchanged gelling temperature of 22°C, mirroring the non-sterile sample. Conversely, dry heat sterilization decreased the gelling temperature to 21°C while gamma irradiation increased it to 24°C. Additionally, pH measures were used to verify whether the hydrogel can be compatible with biological systems (Table 1). Both non-sterile and sterile samples exhibited a neutral pH of around 7. However, ANOVA revealed that steam-heated and irradiated samples reduced the pH with a high statistical significance (pH = 7.01 and 7.00–7.05, respectively, *P *<* *0.0001) compared to the non-sterile sample (pH = 7.20). Notably, following gamma irradiation, a significant decrease (*P *<* *0.0001) in pH was observed, reaching a value of 4.65 compared to all the other samples.

**Table 1.** Gelling properties and pH of non-sterile and sterilized 30%w/v Poloxamer 407 hydrogels, *n* = 3

Sample	Gelling time^a^ (min)	Gelling temperature^a^ (°C)	pH
Non-sterile	2	22	7.20 ± 0.03
Steam heat	2	22	7.01 ± 0.01^b^
Dry heat	2	21	7.17 ± 0.01
Gamma 25 kGy	2	24	4.65 ± 0.01^b^
e-beam 15 kGy	2	22	7.05 ± 0.02^b^
e-beam 25 kGy	2	22	7.00 ± 0.01^b^

a The three replicates yielded identical values.

b Statistically significant difference (*P *<* *0.05) compared to the non-sterile sample (one-way ANOVA).

### Fourier-transform infrared spectroscopy

**Figure 2.**
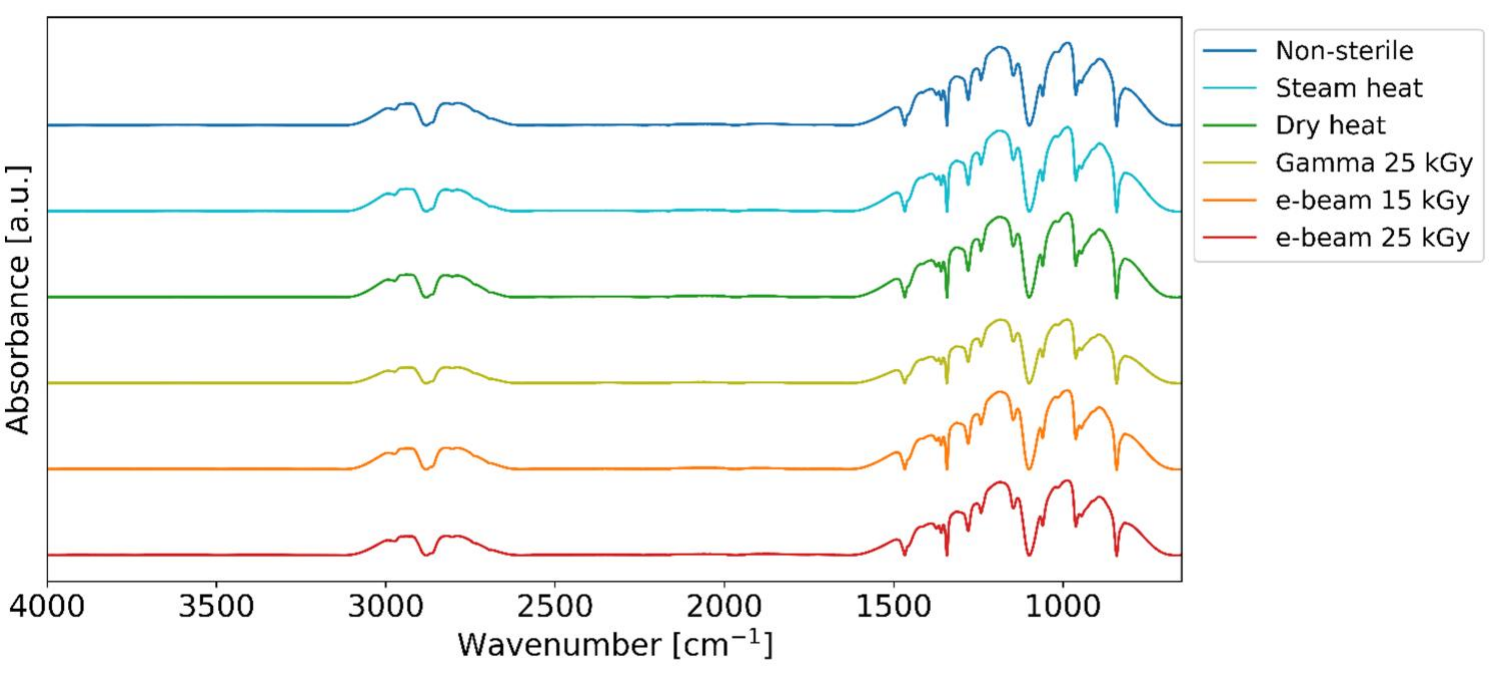
FTIR Spectra of non-sterile and sterilized 30% w/v Poloxamer 407 hydrogels in the 4000–650 cm^−1^ range.

We used FTIR to characterize the chemical properties of the hydrogels before and after sterilization. Figure 2 displays the spectra within the 4000–650 cm^−1^ range. Despite the sterilization process, both sterile and non-sterile hydrogels exhibited similar FTIR profiles. Peaks corresponding to bending vibrations of CH groups (2939–2788 cm^−1^), bending vibrations of CC groups (1491–1389 cm^−1^), bending vibrations of OH groups (1313–1254 cm^−1^) and stretching vibrations of CO groups (1188–985 cm^−1^) were identified in all samples [49, 50]. This denotes the negligible impact of sterilization on the molecular structure of the Poloxamer 407 component.

### Swelling study

**Figure 3.**
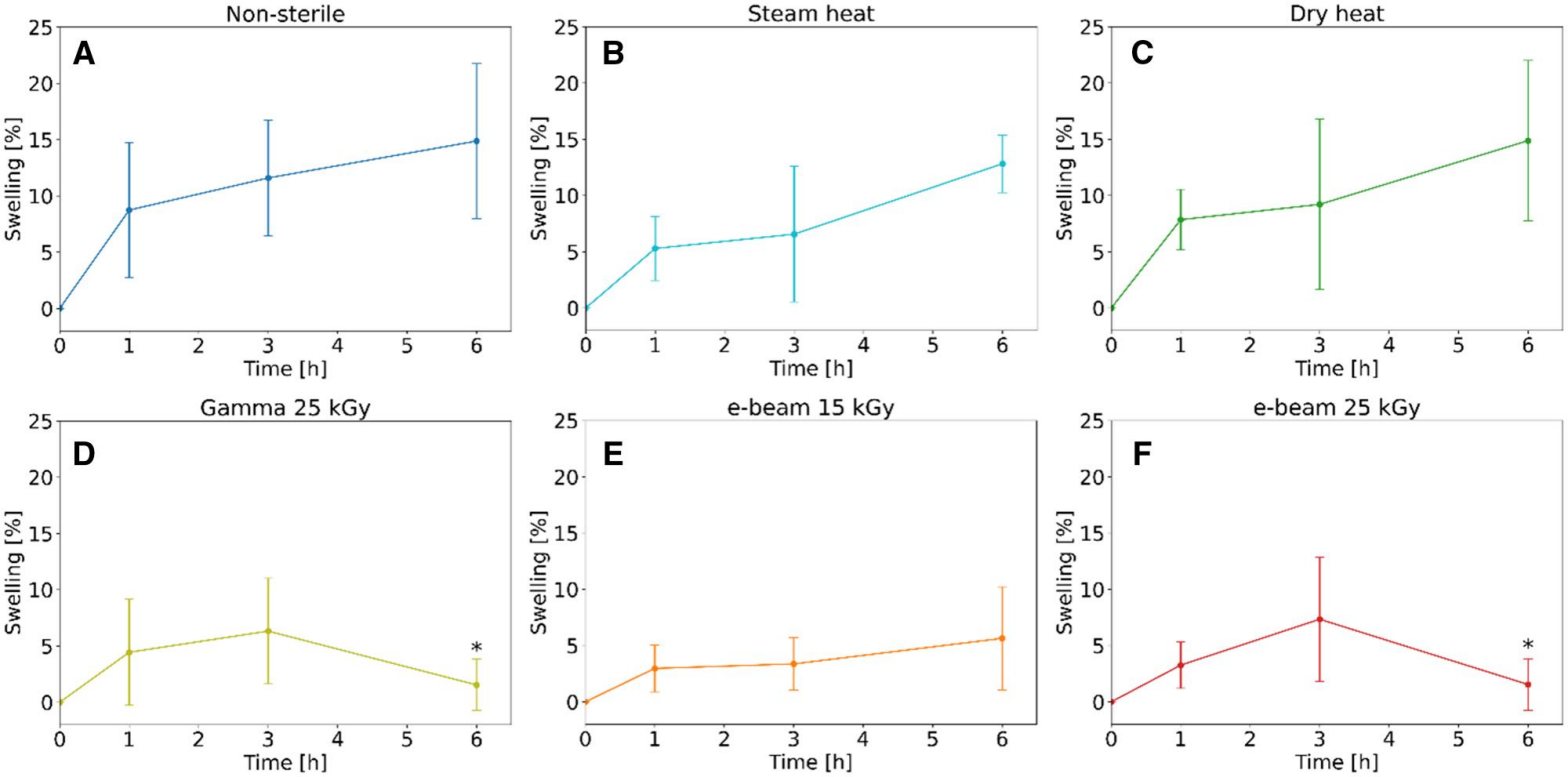
Swelling study of non-sterile and sterilized 30% w/v Poloxamer 407 hydrogels. Mean ± SD, *n* = 5. *Statistically significance difference (*P *<* *0.05) compared to the non-sterile sample (one-way ANOVA). (**A**) Non-sterile, (**B**) steam heat, (**C**) dry heat, (**D**) gamma 25 kGy, (**E**) e-beam 15 kGy, and (**F**) e-beam 25 kGy.

The swelling study results depicted in Figure 3 show that, at the 6-h time point, non-sterile (Figure 3A), steam-heated (Figure 3B) and dry-heated (Figure 3C) samples exhibit greater swelling compared to gamma (Figure 3D) and e-beam (Figure 3E and F) sterilized samples. Furthermore, the swelling of non-sterile, steam-heated and dry-heated samples showed a continual and progressive increase over time, indicating a sustained swelling effect. In contrast, gamma and e-beam sterilized samples maintained relatively stable swelling levels, approximately equivalent to those observed in the first hour, throughout the entire 6-h period. Notably, the *t*-test revealed that the swelling percentage between the initial (*t* = 0) and final (*t* = 6 h) time points was significantly higher (*P *<* *0.05) in both the non-sterile sample and those sterilized by steam heat, dry heat and e-beam at a dose of 15 kGy. However, this change was not statistically significant in the samples sterilized by gamma and e-beam irradiation at a dose of 25 kGy. Moreover, ANOVA shows no statistical differences among any of the groups at the 1- and 3-h time points. However, by the 6-h time point, the swelling percentage in samples sterilized by gamma and e-beam irradiation at a dose of 25 kGy is notably diminished (*P *<* *0.05) compared to the non-sterile, steam-heated, and dry-heated samples.

### Rheology

**Figure 4.**
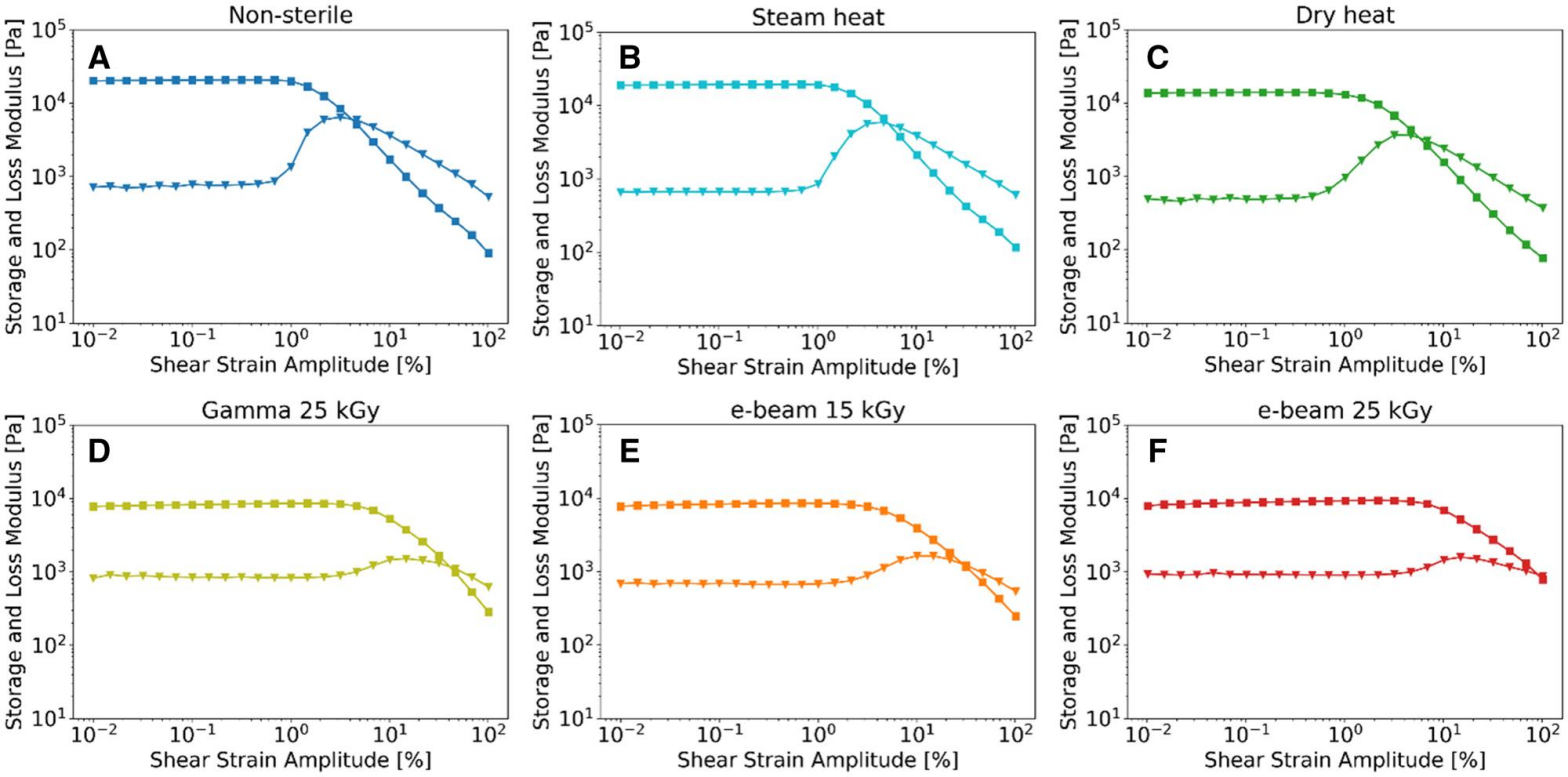
Amplitude sweeps of non-sterile and sterilized 30%w/v Poloxamer 407 hydrogels at 37°C. ■ storage modulus, ▼ loss modulus. (**A**) Non-sterile, (**B**) steam heat, (**C**) dry heat, (**D**) gamma 25 kGy, (**E**) e-beam 15 kGy and (**F**) e-beam 25 kGy.

Rheological measurements were used to investigate the mechanical properties of the hydrogels. In amplitude sweeps conducted at 37°C (Figure 4), non-sterile (Figure 4A), steam-heated (Figure 4B) and dry-heated (Figure 4C) samples displayed storage moduli in the linear viscoelastic region of approximately 20, 19 and 14 kPa, respectively. Notably, the storage modulus decreased to roughly 8 kPa in gamma (Figure 4D) and e-beam (Figure 4E and F) sterilized samples. Moreover, non-sterile, steam-heated and dry-heated samples exhibited a more confined linear viscoelastic region compared to gamma and e-beam irradiated samples. These findings suggest that irradiation reduces rigidity while increasing flexibility, thus enabling more extensive deformation under mechanical forces.

**Figure 5.**
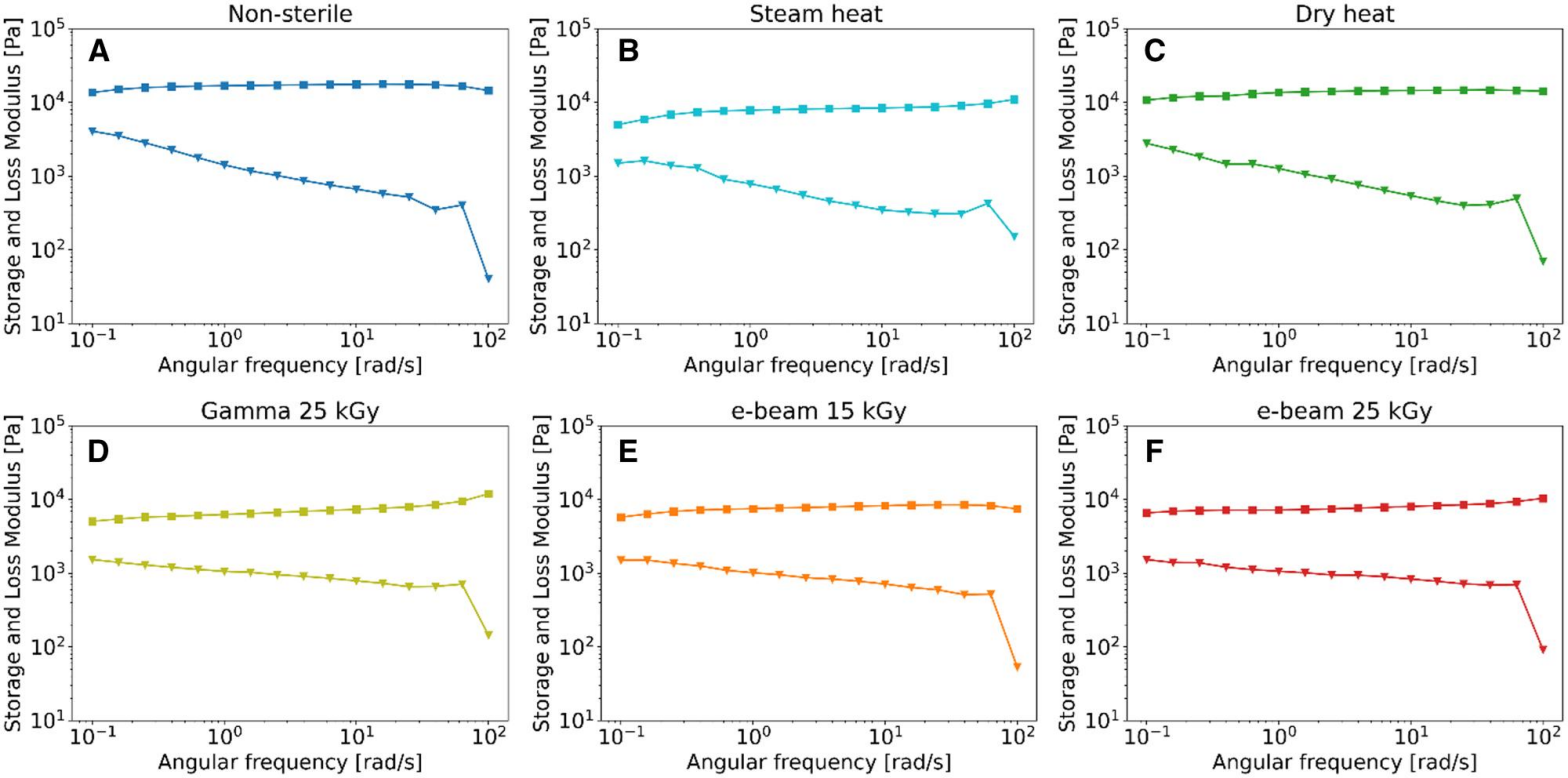
Frequency sweeps of non-sterile and sterilized 30%w/v Poloxamer 407 hydrogels at 37°C. ■ storage modulus, ▼ loss modulus. (**A**) Non-sterile, (**B**) steam heat, (**C**) dry heat, (**D**) gamma 25 kGy, (**E**) e-beam 15 kGy and (**F**) e-beam 25 kGy.

All hydrogels displayed similar behaviour in the frequency sweeps at 37°C (Figure 5), demonstrating a nearly constant storage modulus across the entire angular frequency range and a decreasing loss modulus at elevated frequencies. This hints at a stable mechanical performance in different dynamic conditions.

### Gel permeation chromatography

**Figure 6.**
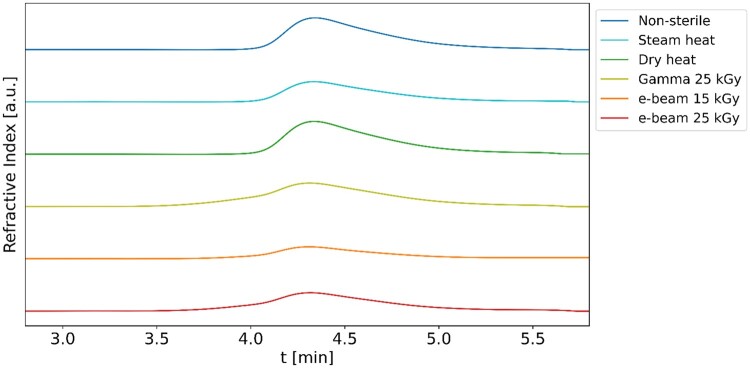
GPC of non-sterile and sterilized 30% w/v Poloxamer 407 hydrogels.

The GPC chromatograms of the samples are presented in Figure 6, showing that the irradiated hydrogels exhibit a broader distribution compared to the non-sterile and heat-sterilized samples. A standard curve was constructed using PEG standards, enabling the calculation of the number average molecular weight (*M_n_*), weight average molecular weight (*M_w_*), peak molecular weight (*M_p_*), Z average molecular weight (*M_z_*) and polydispersity index (PDI) [51]. The calculated molecular weights and PDI values are reported in Table 2.

**Table 2.** Number average molecular weight (*M_n_*), weight average molecular weight (*M_w_*), peak molecular weight (*M_p_*), Z average molecular weight (*M_z_*) and polydispersity index (PDI) of non-sterile and sterilized 30% w/v Poloxamer 407 as calculated from GPC data, mean ± SD, *n* = 3

Sample	*M_n_* (kDa)	*M_w_* (kDa)	*M_p_* (kDa)	*M_z_* (kDa)	PDI
Non-sterile	9.20 ± 0.25	11.68 ± 0.37	12.05 ± 0.40	13.40 ± 0.46	1.269 ± 0.007
Steam heat	9.34 ± 0.26	11.99 ± 0.39	12.31 ± 0.23	13.89 ± 0.50	1.285 ± 0.008
Dry heat	9.26 ± 0.21	11.77 ± 0.29	12.18 ± 0.23	13.51 ± 0.33	1.272 ± 0.002
Gamma 25 kGy	11.08 ± 0.09^a^	15.04 ± 0.12^a^	12.73 ± 0.24	17.91 ± 0.11^a^	1.357 ± 0.0003^a^
e-beam 15 kGy	10.34 ± 0.09^a^	13.99 ± 0.31^a^	12.86 ± 0.02	16.62 ± 0.39^a^	1.353 ± 0.018^a^
e-beam 25 kGy	10.94 ± 0.18^a^	14.69 ± 0.23^a^	12.73 ± 0.48	17.45 ± 0.20^a^	1.342 ± 0.008^a^

a Statistically significant difference (*P *<* *0.05) compared to the non-sterile sample (one-way ANOVA).

The irradiated samples showed a significant increase in *M_n_*, *M_w_*, *M_z_* and PDI (*P* ≤ 0.0001) when compared to the non-sterile and heat-sterilized samples. However, no significant difference was observed among the groups in terms of *M_p_*. Both e-beam and gamma irradiation increased the molecular weight, though the e-beam irradiation at 15 kGy demonstrated a lower impact compared to gamma irradiation, with significantly lower values for *M_n_* (*P *=* *0.0053), *M_w_* (*P *=* *0.0106) and *M_z_* (*P *=* *0.0089).

### Small-angle X-ray scattering

**Figure 7.**
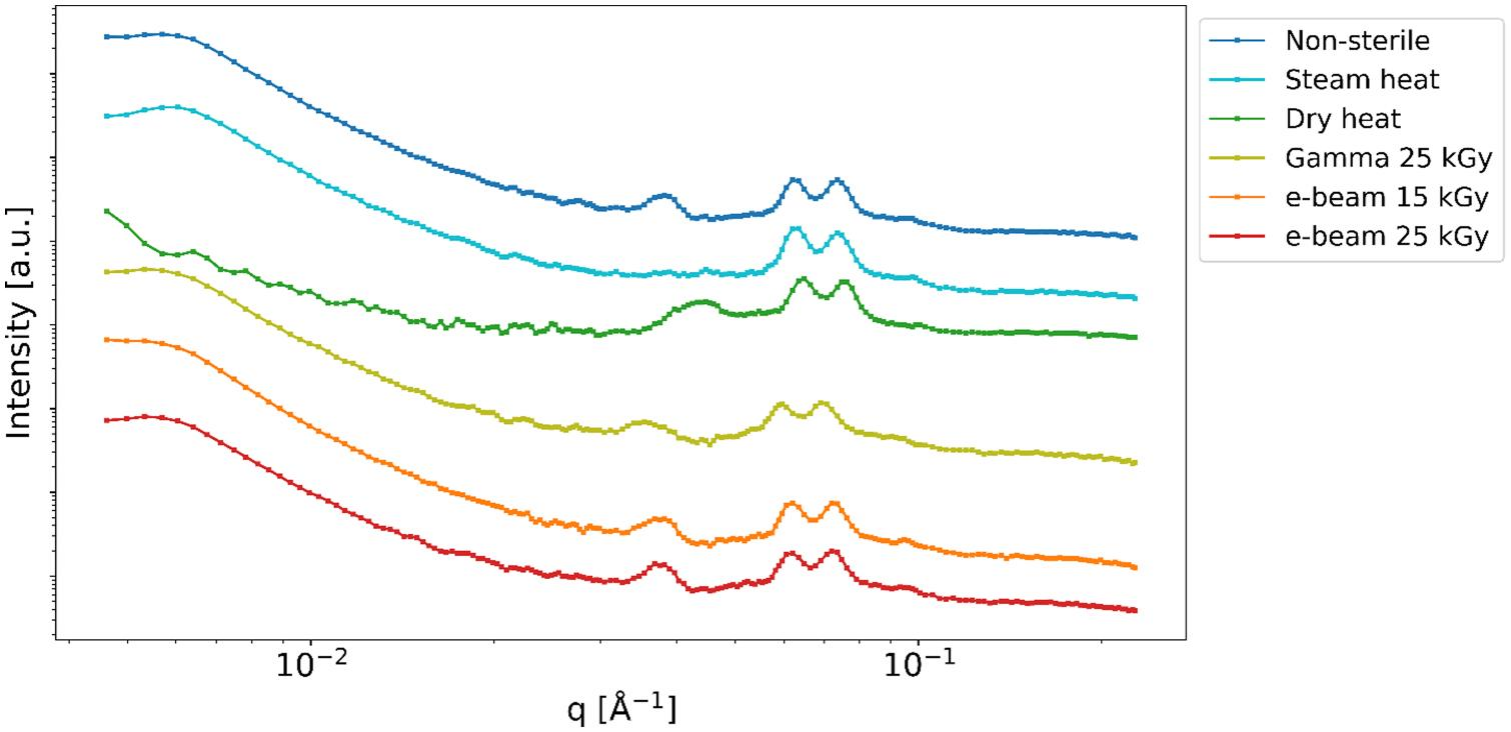
SAXS spectra of non-sterile and sterilized 30% w/v Poloxamer 407 hydrogels.

The SAXS spectra are presented in Figure 7. To determine the packing structure, the first-order peak position *q_1_* was identified, and the ratios of the subsequent peaks were evaluated. The results indicate local hexagonal ordering for all the samples, where the q-positions of the diffraction peaks are in the ratios of 1:√3:√4:√7. However, the steam-heated hydrogel does not display the first-order peak *q_1_*. A hexagonal phase has Bragg peaks at:


(1) $$q=\frac{4\pi }{a\surd 3}\cdot \sqrt{h^{2}+hk+k^{2},}$$

where *a* is the lattice parameter, i.e. the unit cell side length, and *h* and *k* are the Miller indexes [52, 53]. Since *q_1_* is absent in the steam-heated sample, the lattice parameter for the hexagonal structure was calculated based on the second-order peak *q_2_*, corresponding to the Miller indices *h *=* k *=* *1:


(2) $$a=\frac{4\pi }{q_{2}}$$

The lattice parameter values are reported in Table 3. The non-sterile, steam-heated and e-beam irradiated samples at 15 kGy show the exact lattice parameter value (*a *=* *19.77 nm). In contrast, there is a slight increase in the gamma-irradiated (*a *=* *20.73 nm) and e-beam irradiated samples at 25 kGy (*a *=* *20.27 nm) and a slight decrease in the dry-heated sample (*a *=* *19.35 nm).

**Table 3.** Lattice parameter (*a*) for the hexagonal structure of non-sterile and sterilized 30% w/v Poloxamer 407 hydrogels as calculated from SAXS data, *n* = 1

Sample	*a* (nm)
Non-sterile	19.77
Steam heat	19.77
Dry heat	19.35
Gamma 25 kGy	20.73
e-beam 15 kGy	19.77
e-beam 25 kGy	20.27

### LDH assay

**Figure 8.**
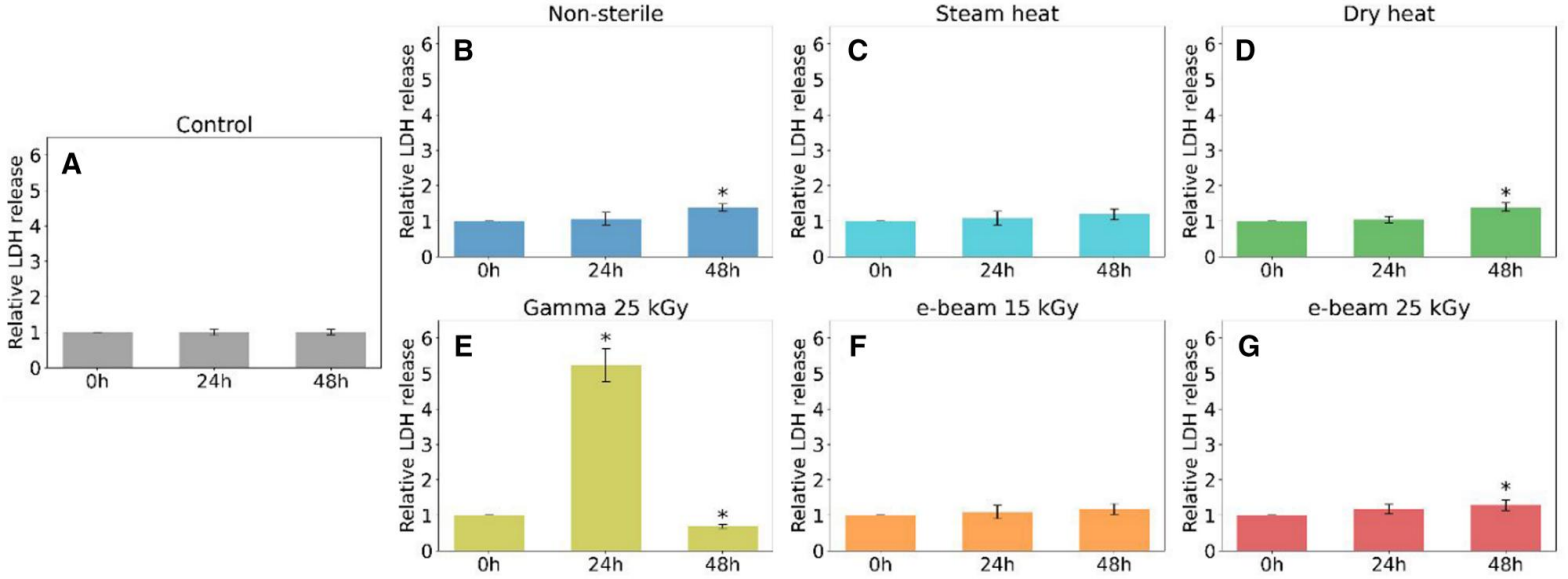
Relative LDH release from non-sterile and sterilized 30% w/v Poloxamer 407 hydrogels. Mean ± SD, *n* = 3; *Statistical significance (*P *<* *0.05) compared to the initial time point (*t*-test). (**A**) control, (**B**) non-sterile, (**C**) steam heat, (**D**) dry heat, (**E**) gamma 25 kGy, (**F**) e-beam 15 kGy and (**G**) e-beam 25 kGy.

**Figure 9.**
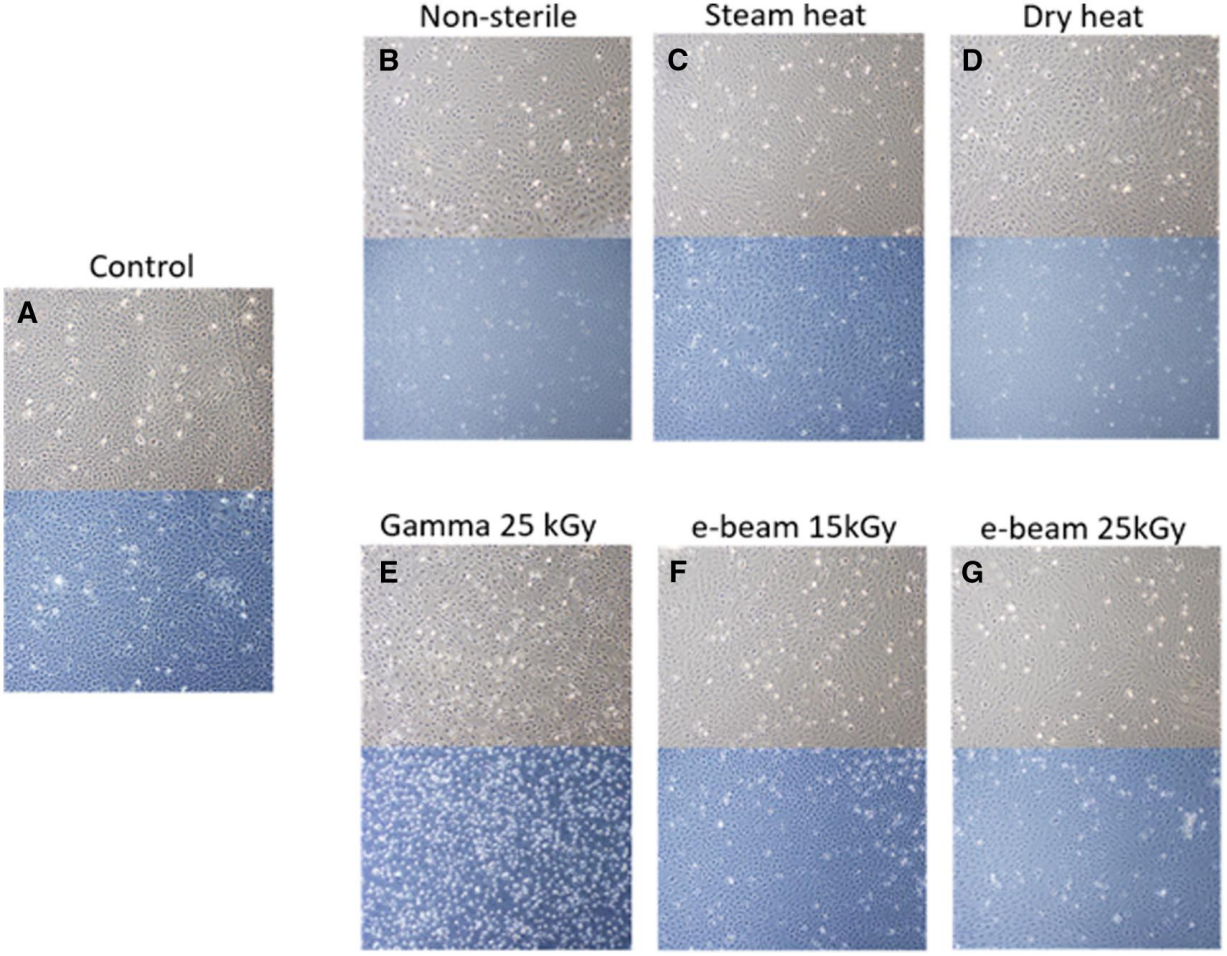
Cells morphology in non-sterile and sterilized 30% w/v Poloxamer 407 hydrogels. Upper panel: 24-h time point. Lower panel: 48-h time point. (**A**) control, (**B**) non-sterile, (**C**) steam heat, (**D**) dry heat, (**E**) gamma 25 kGy, (**F**) e-beam 15 kGy and (**G**) e-beam 25 kGy.

The LDH Assay was used to assess the cytotoxicity of the hydrogels. The relative LDH release at 24 and 48 h is presented in Figure 8, while Figure 9 displays the corresponding cell images. Notably, the gamma-sterilized sample (Figure 8E) exhibited a substantial increase in relative LDH release after 24 h (*P *<* *0.001), followed by a decrease at 48 h (*P *=* *0.002). The intergroup comparison performed with ANOVA revealed that the LDH release from the gamma-irradiated sample is significantly different (*P *<* *0.05) than that of the other samples at both 24 and 48 h, with no significant difference between the other groups. Figure 9E illustrates evident cell death in the gamma-sterilized sample at 24 h, characterized by cell shrinkage and detachment, with an even more pronounced effect at 48 h.

Furthermore, an increase in relative LDH release after 48 h was noted in non-sterile (*P *=* *0.003), dry-heated (*P *=* *0.003) and e-beam 25 kGy (*P *=* *0.024) sterilized samples, although the variation was slight. This increase was not significant in the steam-heated and e-beam 15 kGy irradiated samples. All hydrogels exhibited a subtle alteration in cell morphology after 48 h, yet the change was notably significant in the gamma-sterilized sample already after 24 h.

### Determination of peroxide groups

**Figure 10.**
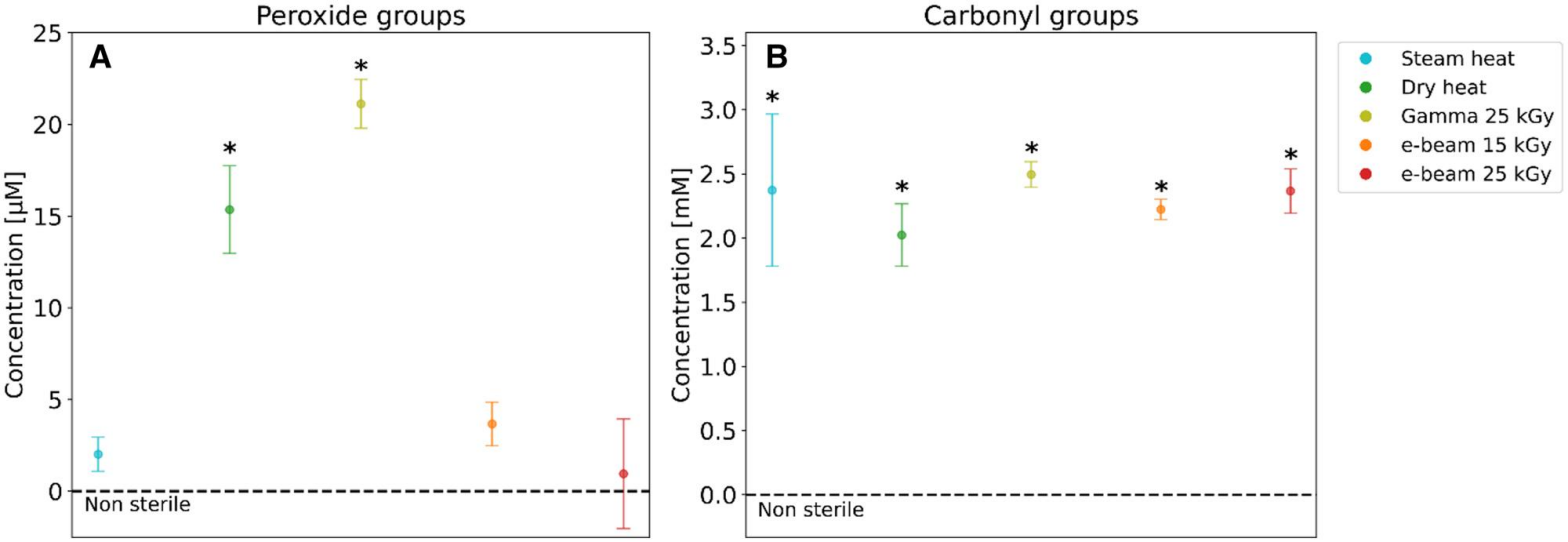
(**A**) Peroxide groups concentration in sterilized 30% w/v Poloxamer 407 hydrogels, with non-sterile values subtracted, mean ± SD, *n* = 3. (**B**) Carbonyl groups concentration in sterilized 30% w/v Poloxamer 407 hydrogels, with non-sterile values subtracted, mean ± SD, *n* = 3. *A statistically significant difference (*P* < 0.05) compared to the non-sterile sample (one-way ANOVA).

The presence of peroxide groups, indicative of oxidative degradation, was assessed using the Pierce^TM^ Quantitative Peroxide Assay Kit [54, 55]. Blank values and non-sterile sample values were subtracted to isolate the peroxide groups formed due to sterilization. The results presented in Figure 10A show that hydrogels subjected to dry heating (*P *<* *0.0001) and gamma irradiation (*P *<* *0.0001) exhibit a significantly higher concentration of peroxide groups compared to non-sterile, steam-heated, and e-beam irradiated hydrogels. Notably, gamma irradiation results in the highest concentration of peroxide groups, surpassing even the dry-heated sample (*P *=* *0.0179).

### Determination of carbonyl groups

The presence of carbonyl groups, indicative of oxidative degradation, was measured using a MAK486 Carbonyl Assay Kit [54, 55]. The measured values were multiplied by 20 to account for the 1:20 dilution of the samples. Additionally, blank values and non-sterile sample values were subtracted to isolate the carbonyl groups formed due to sterilization. As shown in Figure 10B, all sterilized hydrogels exhibited a significant increase in carbonyl concentration (*P* < 0.0001) compared to the non-sterile sample. However, no significant differences were observed among the sterilized groups.

## Discussion

Poloxamer 407 hydrogels are of growing interest in the cosmetic, biomedical and pharmaceutical fields due to their thermo-sensitive characteristics, enabling the formation of injectable products that gel *in situ* at body temperature [15, 56]. Sterilization, a crucial prerequisite in clinical applications, has been thoroughly investigated in this study regarding its effects on the functional properties of a 30% w/v Poloxamer 407 hydrogel formulation. Specifically, sterilization was performed by steam heat (121°C, 20 min), dry heat (160°C, 1 h), gamma irradiation (25 kGy) and e-beam irradiation (15 kGy and 25 kGy).

All the tested gels, including a non-sterile sample, gelled within 2 min (Table 1). However, the actual gelation time may be shorter, as this interval represents the minimum detectable time due to the experimental setup, which ensures homogeneous heating throughout the sample. Notably, the dry-heated sample gelled at a temperature of 21°C, i.e. 1°C lower than the non-sterile gel. This significant difference was likely due to water evaporation during the dry heat process, which increased the Poloxamer 407 concentration and thus lowered the gelling temperature. This effect was not observed in the steam-heated sample, with milder conditions.

Previous research by Beard *et al.* indicated that autoclaving Poloxamer 407 hydrogels can cause water evaporation, particularly in gels with lower polymer concentrations [42]. However, the gelation temperature of 30% w/v and 40% w/v formulations was not significantly affected, with only the 35% w/v formulation showing a reduction [42].

Rafael *et al*. noted a modest increase in gelation time for a 20% w/v Poloxamer 407 gel post-autoclaving [43]. At the same time, dry heat sterilization led to a loss of gelation properties due to thermal-oxidative degradation, the latter being confirmed by an increase of reactive oxygen species [43]. In the present study, while the dry-heated hydrogel retained its gelation properties, it showed a significantly higher concentration of peroxide groups (Figure 10A), aligning with Rafael *et al*.’s findings, albeit at a lower magnitude, which is a sign of oxidative degradation. Furthermore, a notable increase in carbonyl groups (Figure 10B) was observed in all the sterilized hydrogels, indicating oxidative degradation across all treatments. Conversely, Ferreira *et al.* reported a minor decrease in both gelation temperature and time after autoclaving a 20% w/v Poloxamer 407 hydrogel, hinting at water evaporation, with no signs of degradation [35]. In addition, Gupta and colleagues found that autoclaving did not affect the pH, gelling properties or viscosity of a Poloxamer 407 and chitosan hydrogel formulation to release timolol maleate [57]. This aligns with studies by Jansen *et al.* and Zanon *et al.*, which demonstrated the effectiveness of autoclaving Poloxamer 407 formulations for drug delivery [58, 59]. Overall, while heat sterilization tends to cause water evaporation, it is deemed that any alteration in gelation properties relies heavily on the specific equipment, sterilization parameters, and gel formulation used. Since the dry heat process requires harsher conditions, it is more likely to observe an alteration in the gel’s properties.

Regarding irradiation, e-beam sterilized samples maintained the same gelation temperature of 22°C as the non-sterile gel, while gamma irradiation raised the gelation temperature to 24°C, suggesting potential degradation (Table 1) due to its higher penetration ability. This is further supported by a significant decrease in pH after gamma irradiation, resulting in an average value of 4.65, and a substantial increase in peroxides concentration (Table 1 and Figure 10A), which can degrade the hydrogel either immediately or in a subsequent phase. These changes can be ascribed to chain scission and oxidative reactions during the air-atmosphere irradiation [54, 60–62]. Indeed, oxygen generates peroxide radicals that favour chain scission reactions, leading to a greater number of oxygenated species, carbonyl groups (Figure 10B), and ultimately to formic acid and acetic acid [55, 61, 63, 64]. Notably, the increase in peroxide concentration, particularly in the dry heat and gamma-irradiated hydrogels, suggests that degradation reactions are still actively occurring, which could lead to further degradation altering the polymer structure. However, it is important to note that freeze-drying of the samples before FTIR analysis prevented the detection of formic and acetic acids, which are plausibly responsible for the observed pH drop in the gamma-irradiated hydrogel. In contrast, the pH of the other hydrogels remained within the neutral range of 7.0–7.2 (Table 1). Additionally, the GPC elution profiles (Figure 6) revealed a broader distribution of molecular weights and higher polydispersity in the irradiated samples, indicating oxidative degradation as previously observed [64]. Gamma and e-beam irradiation, a complex process with multifaceted effects, can increase polymer molecular weight through crosslinking, where high-energy rays generate free radicals that form covalent bonds between polymer chains [65, 66]. Gamma irradiation can also induce chain scission, reducing molecular weight [65]. The overall effect results from a delicate balance of factors such as polymer type, irradiation dose and oxygen availability. For instance, polydimethylsiloxane and polyurethane exhibited a pronounced increase in molecular weight at specific doses [65–67]. As different polymers vary in susceptibility to crosslinking or chain scission, the net impact of gamma irradiation on molecular weight is highly polymer- and condition-dependent [68–70].

Despite these findings, Nesseem’s study on a sparfloxacin-containing formulation of Pluronic^®^ F127 and F68 did not detect any difference in physical appearance after gamma sterilization [71]. Studies have used electron beam and gamma irradiation to induce crosslinking in Poloxamer 407 formulations with other polymers, but the specific impact of irradiation on the Poloxamer 407 component alone remains unclear [72, 73].

The gels’ FTIR spectra (Figure 2) revealed that the sterilization processes did not alter the Poloxamer 407’s core functional groups, as evidenced by the presence of its characteristic peaks.

This aligns with a previous study on a steam-heated 20% w/v Poloxamer 407 hydrogel [35]. Moreover, the studies by Nesseem and Al Kayal *et al*. reported no discernible alterations in the FTIR spectra following gamma irradiation of a Poloxamer hydrogel [71, 74].

The SAXS spectra (Figure 7) indicate a hexagonal arrangement of the micelles across all samples. However, the steam-heated sample does not exhibit the first-order peak at *q_1_*, possibly due to the superimposition of the form factor (*P*_(*q*)_) minima on the structure factor (*S*_(*q*)_) maxima. Additionally, the dry-heated hydrogel displays a straighter curve than other samples, suggesting that its structure is more homogenous. While a face-centred cubic lattice is typically observed in Poloxamer 407 hydrogels, both hexagonal and cubic structures have been documented in the literature, with variations that might be attributed to differences in Poloxamer concentration, the temperature of analysis, and shear forces in sample preparation [2, 6, 75–78].

The dry-heated sample showed a slight decrease in lattice parameter, indicating a more compact hexagonal structure, consistent with the observed lower gelation temperature and suggesting water evaporation and increased Poloxamer 407 concentration (Table 3). In contrast, this effect was not seen in the steam-heated sample. Gamma and e-beam irradiation at 25 kGy increased the lattice parameter, especially with gamma irradiation, suggesting potential degradation due to chain scission and oxidative reactions. These reactions might alter the hydrophilic-lipophilic balance, potentially resulting in looser micelle packing. This effect was not observed in the e-beam irradiated sample at a dose of 15 kGy.

Moreover, the gamma-irradiated hydrogel exhibited a notably high relative LDH release after 24 h, followed by a decrease after 48 h, suggesting a cytotoxic effect (Figure 8E). The subsequent drop in LDH release is plausibly due to the severe cell mortality already occurring by the 24-h time point, implying that fewer viable cells remain to release LDH at 48 h. This might be ascribed to the acidic pH of the gamma-irradiated gel. In contrast, the other hydrogels demonstrated comparable patterns of LDH release and exhibited only minor cell morphology alterations after 24 and 48 h (Figure 9).

The swelling study revealed similar percentages in the non-sterile, steam-heated and dry-heated samples, characterized by a progressively increasing swelling ratio over time (Figure 3). On the other hand, both gamma and e-beam sterilized samples showed a reduced swelling ratio, with values matching those at the initial time point, even after 6 h. The increase in *M_n_*, *M_w_* and *M_z_* in the irradiated samples (Table 2) suggests that crosslinking has occurred alongside chain scission, aligning with prior observations in biomedical polymers [66, 79]. The swelling behaviour is indeed consistent with the presence of crosslinked networks, which restrict water uptake. These outcomes indicate that sterilization by irradiation might be preferable to heat methods since excessive swelling could dilute the Poloxamer 407, thereby modifying its properties. We selected a 6-h swelling duration to capture the initial swelling dynamics, which was sufficient for observing the key swelling behaviour of the hydrogels. Conversely, the study by Benning *et al.* displays degradation of 15% w/v, 20% w/v and 25% w/v non-sterile Poloxamer 407 hydrogels over 1 week [80]. The contrasting results can be attributed to the differences in Poloxamer 407 concentrations and experimental methodologies. Benning *et al.* employed longer incubation times and immediately added endothelial cell growth medium to their formulations when they achieved a semi-solid state of the samples, potentially accelerating degradation. Moreover, the medium contains multiple biologically active components that might interact with and degrade the hydrogel matrix. On the other hand, in the present study, we used DPBS as a swelling agent, a more straightforward and more inert solution, after the samples were allowed to stabilize in a gel state for 15 min at 37°C. The use of DPBS and the timing of its addition may have contributed to a more controlled swelling and absence of degradation. Additionally, the higher concentration of Poloxamer 407 used in our study likely resulted in a more robust gel network that is less susceptible to degradation under these conditions and timespan.

The amplitude sweeps showed that non-sterile, steam-heated and dry-heated samples possess similar properties, corroborated by research from Beard *et al.*, which indicates that autoclaving generally does not alter the rheological features of Poloxamer 407 hydrogels with concentrations of 30% w/v or more [42]. Specifically, non-sterile, steam-heated and dry-heated hydrogels exhibited higher storage (*G′*) modulus compared to irradiated samples, yet the irradiated gels presented a crossover point shifted at higher shear strain amplitudes, revealing a considerably more extended linear viscoelastic region, i.e. a higher resistance to deformation (Figure 4).

The reduction in *G′* after irradiation is likely associated with chain scission reactions, producing smaller polymer fragments, which typically lead to a decrease in the density of chain entanglements, consequently diminishing both the *G*′ and *G*″ values [81]. However, the resultant shorter chains possess increased flexibility, facilitating more rapid entanglement and disentanglement. This enhanced mobility allows the entanglement density to remain constant over an extended range of shear rates. Additionally, shorter polymer chains can result in a more gradual increase in *G″* before the crossover point for irradiated samples compared to non-sterile and heat-sterilized samples due to the fewer and weaker entanglements. At the same time, the improved resistance to deformation of the irradiated samples aligns with the observed molecular weight increases (*M_n_*, *M_w_* and *M_z_*), an indication of crosslinking reactions. Hence, the combination of chain scission and crosslinking can explain the rheological behaviour of the irradiated hydrogels, characterized by reduced rigidity yet improved resistance to deformation (Figure 4).

Furthermore, the frequency sweeps showed analogous trends across all gel types, with a nearly constant *G′* and a decreasing *G″* across the frequency range (Figure 5), which is in agreement with other studies on non-sterile Poloxamer 407 hydrogels [82, 83]. *G′* exceeding *G″* across the full range of frequencies confirmed the hydrogels’ stability. Notably, *G″* of irradiated hydrogels does not decrease as steeply as the non-sterile and heat-sterilized samples, indicating a less pronounced rigid response across the frequency range. This is plausibly due to the presence of shorter polymer chains, which facilitate mobility and result in greater internal friction and energy loss. However, at high frequency (100 rad/s), all samples exhibit a sudden decrease in *G″*, as rapid oscillations restrict the polymer chains’ ability to respond to the applied stress, leading to limited chain mobility, reduced energy dissipation, and a more solid-like behaviour. Notably, a rough parallel plate was not available for the rheological analyses due to experimental limitations, which may lead to wall slip [84]. To address this concern, the frequency sweep was conducted as a small amplitude oscillatory test (0.05%), helping to preserve the hydrogel's structural integrity and minimize flow at the wall interface.

Moreover, the soft solid state of heated Poloxamer 407 hydrogels is often termed the gel state; however, it is essential to differentiate between the gel and glassy states within this phase. Suman *et al.* propose a phase transition mechanism where the glassy state follows the gel state with increasing temperature, characterized by larger micelles, denser packing, and higher rigidity [85]. According to this mechanism, a 30% w/v Poloxamer 407 hydrogel at 37°C exhibits a glassy state. This study chose the 37°C temperature to simulate the body temperature for clinical applications.

## Conclusion

This study aimed to identify a sterilization method that effectively preserves the functional properties of a 30% w/v Poloxamer 407 hydrogel formulation by assessing the effects of various heat and radiation techniques. Key findings include that steam heat and electron beam (e-beam) sterilization maintained the gelling temperature at 22°C, mirroring the non-sterile sample. In contrast, dry heat decreased the gelling temperature while gamma irradiation increased it. All the sterilized hydrogels showed an increased concentration of carbonyl groups, suggesting potential degradation. Additionally, dry heat and gamma irradiation resulted in a higher concentration of peroxide groups than the other hydrogels, indicating that degradation reactions are still actively occurring. GPC revealed a broader molecular weight distribution, higher polydispersity in the irradiated hydrogels, and an increase of *M_n_*, *M_w_* and *M_z_*, highlighting the interplay between crosslinking and chain scission reactions. Gamma irradiation also significantly reduced the pH to 4.65, further supporting degradation. Conversely, other sterilization methods preserved a neutral pH. However, sterilization did not alter the core functional groups of Poloxamer 407 in any of the samples, as confirmed by FTIR analysis. Swelling behaviour revealed that non-sterile, steam-heated and dry-heated samples exhibited progressive swelling over time, while gamma and e-beam sterilized samples showed stable swelling ratios, with the 25 kGy irradiated samples demonstrating the slightest swelling. Rheological properties indicated that non-sterile, steam-heated and dry-heated samples had higher storage moduli than irradiated samples; however, irradiated hydrogels exhibited a more extended linear viscoelastic region, indicating enhanced resistance to deformation. Cytotoxicity assessments showed that gamma-sterilized hydrogels were cytotoxic, evidenced by increased LDH release and significant cell death. In contrast, heat- and e-beam sterilized samples maintained low cytotoxicity comparable to non-sterile hydrogels. Based on these results, autoclaving preserves the hydrogel properties but fails to improve them, which is a limitation since excessive swelling might affect its suitability for clinical use. Hence, e-beam irradiation within the 15–25 kGy range emerges as the most effective sterilization method for Poloxamer 407 hydrogels, preserving and significantly enhancing their desirable properties. Eventually, the choice of the sterilization method should also be guided by the cost-effectiveness and the criticality of the hydrogel’s properties in a specific application.

Future investigations should explore a broader range of Poloxamer 407 concentrations to expand the applicability of these findings in diverse clinical uses. Further studies on long-term stability under real storage conditions are also recommended to verify the sustained functionality and sterility of the hydrogels over time. Ultimately, when progressing toward clinical application, rigorous validation of the sterilization process in full compliance with healthcare product standards is essential.
